# Synergistic silencing of α-globin and induction of γ-globin by histone deacetylase inhibitor, vorinostat as a potential therapy for β-thalassaemia

**DOI:** 10.1038/s41598-019-48204-2

**Published:** 2019-08-12

**Authors:** Sachith Mettananda, Nirmani Yasara, Christopher A. Fisher, Stephen Taylor, Richard Gibbons, Doug Higgs

**Affiliations:** 1 Department of Paediatrics, University of Kelaniya, Thalagolla Road, Ragama, 11010 Sri Lanka; 2 Medical Research Council (MRC) Molecular Haematology Unit, Weatherall Institute of Molecular Medicine, University of Oxford, John Radcliffe Hospital, Headington, Oxford, OX3 9DS UK; 3 Weatherall Institute of Molecular Medicine, University of Oxford, Oxford, UK

**Keywords:** Molecular medicine, Anaemia

## Abstract

β-Thalassaemia is one of the most common monogenic diseases with no effective cure in the majority of patients. Unbalanced production of α-globin in the presence of defective synthesis of β-globin is the primary mechanism for anaemia in β-thalassaemia. Clinical genetic data accumulated over three decades have clearly demonstrated that direct suppression of α-globin and induction of γ-globin are effective in reducing the globin chain imbalance in erythroid cells hence improving the clinical outcome of patients with β-thalassaemia. Here, we show that the histone deacetylase inhibitor drug, vorinostat, in addition to its beneficial effects for patients with β-thalassaemia through induction of γ-globin, has the potential to simultaneously suppress α-globin. We further show that vorinostat exhibits these synergistic beneficial effects in globin gene expression at nanomolar concentrations without perturbing erythroid expansion, viability, differentiation or the transcriptome. This new evidence will be helpful for the interpretation of existing clinical trials and future clinical studies that are directed towards finding a cure for β-thalassaemia using vorinostat.

## Introduction

β-Thalassaemia is one of the most common genetic diseases with over 70,000 new patients diagnosed throughout the world every year^1^. The majority of these births occur in the traditional thalassaemia belt which extends from the Mediterranean through the middle east and sub-Saharan Africa to south and southeast Asia^2^. Nevertheless, due to continued migration, β-thalassaemia has more recently become a significant health problem in many developed countries in Europe, Australia and North America^3^.

The clinical management of β-thalassaemia still largely relies on supportive care with regular lifelong blood transfusions and iron chelation^4–7^. The only current effective cure is allogenic stem cell transplantation, however, this is not available to most patients due to the lack of suitable donors and the associated cost of transplantation^8^. Therefore, even in developed countries, most patients with β-thalassaemia are managed exclusively using supportive care and, consequently, they experience shorter life expectancies and premature deaths^9^.

The molecular defects causing β-thalassemia are point mutations predominantly found within and around the β-globin gene which result in reduced or absent synthesis of β-globin^10^. This unbalanced production of α- and β-like globin chains leads to precipitation of free α-globin chains in red blood cells (RBC) and their precursors to cause haemolysis and ineffective erythropoiesis which are considered as the primary pathophysiological mechanism for the anaemia in thalassaemia^11^. The clinical and genetic data accumulated over several decades through naturally occurring human mutations have identified, two independent pathways that could counter-act the globin imbalance in order to ameliorate the severity of β-thalassaemia^12^. Firstly, the induction of γ-globin increases the β-like globin chains in the RBC and “mops-up” excess α-globin chains by producing fetal haemoglobin, haemoglobin F (HbF)^13,14^. Secondly, direct silencing of α-globin has shown to ameliorate the severity of β-thalassaemia considerably^12,15^. Both of these mechanisms have been exploited independently, using various experimental pharmacological and genetic-based therapies, to identify a cure for β-thalassaemia however, this has met variable success so far^16–19^. In this paper, we depict the synergistic use of both these mechanisms by the US FDA approved histone deacetylase (HDAC) inhibitor drug, vorinostat. We show that vorinostat directly reduces the production of α-globin whilst inducing the expression of γ-globin in human erythroid cells without perturbing erythroid proliferation, viability, differentiation or global gene expression.

## Results

### Effect of HDAC inhibitors on globin gene expression in human erythroid cells

**Figure 1:**
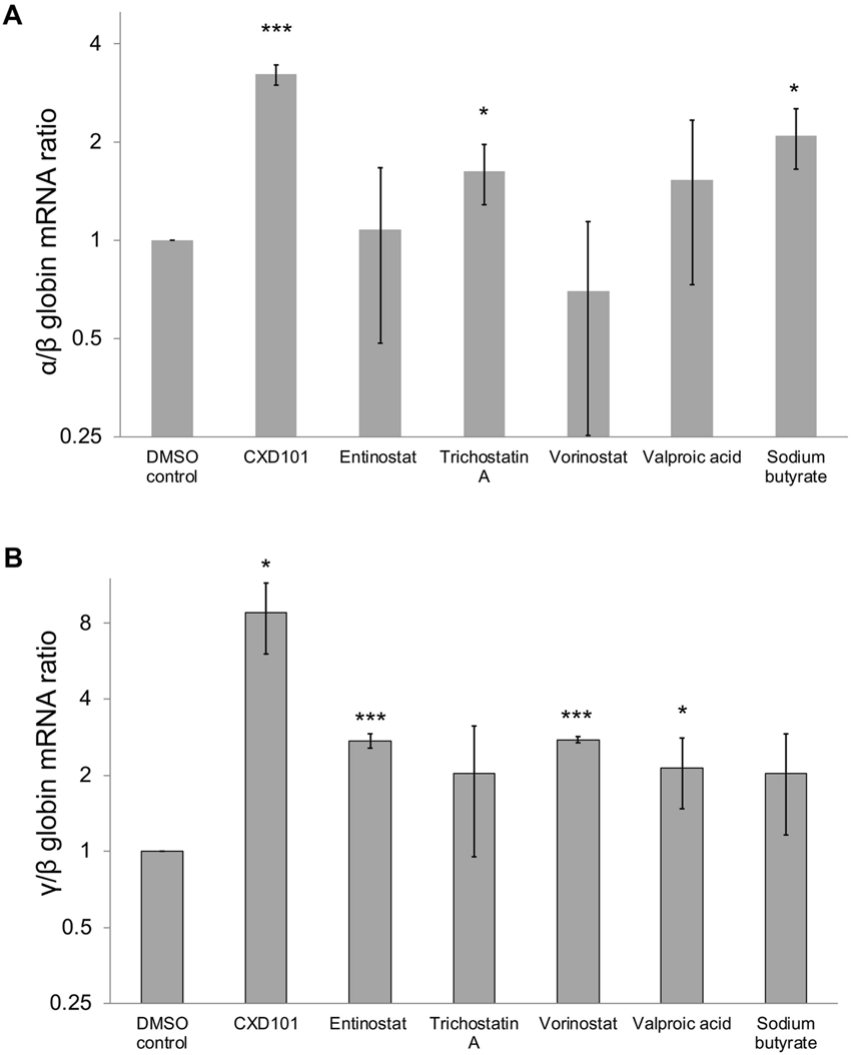
(**A**) α/β and (**B**) γ/β mRNA ratios of erythroid cells treated with HDAC inhibitors. Mean ratios referenced to DMSO control from 3 independent biological repeats of erythroid cells differentiated from human umbilical cord blood CD34^+^ cells are shown. Error bars represent SD; *p < 0.05 and ***p < 0.001. Scale of the y-axis is logarithmic (base-2).

We previously performed a small molecule screen using Fluidigm high throughput qPCR platform to identify compounds that alter globin gene expression in human erythroid cells to identify compounds that produce favourable effects for β-thalassaemia. This screen identified several promising compounds that down regulate α-globin expression of which IOX1 (also known as 5-carboxy-8-hydroxyquinoline), a broad-spectrum histone demethylase inhibitor was studied in depth^17^. Subsequent analysis of this small molecule screen identified a number of histone deacetylase (HDAC) inhibitor drugs which upregulate γ-globin expression in human erythroid cells (Supplemental Table 1). One of these, vorinostat, was particularly important as it also down regulated α-globin expression (although not statistically significant in this less sensitive assay) whilst upregulating the expression of γ-globin (Fig. 1A,B).

### Effects of vorinostat on globin gene expression

**Figure 2:**
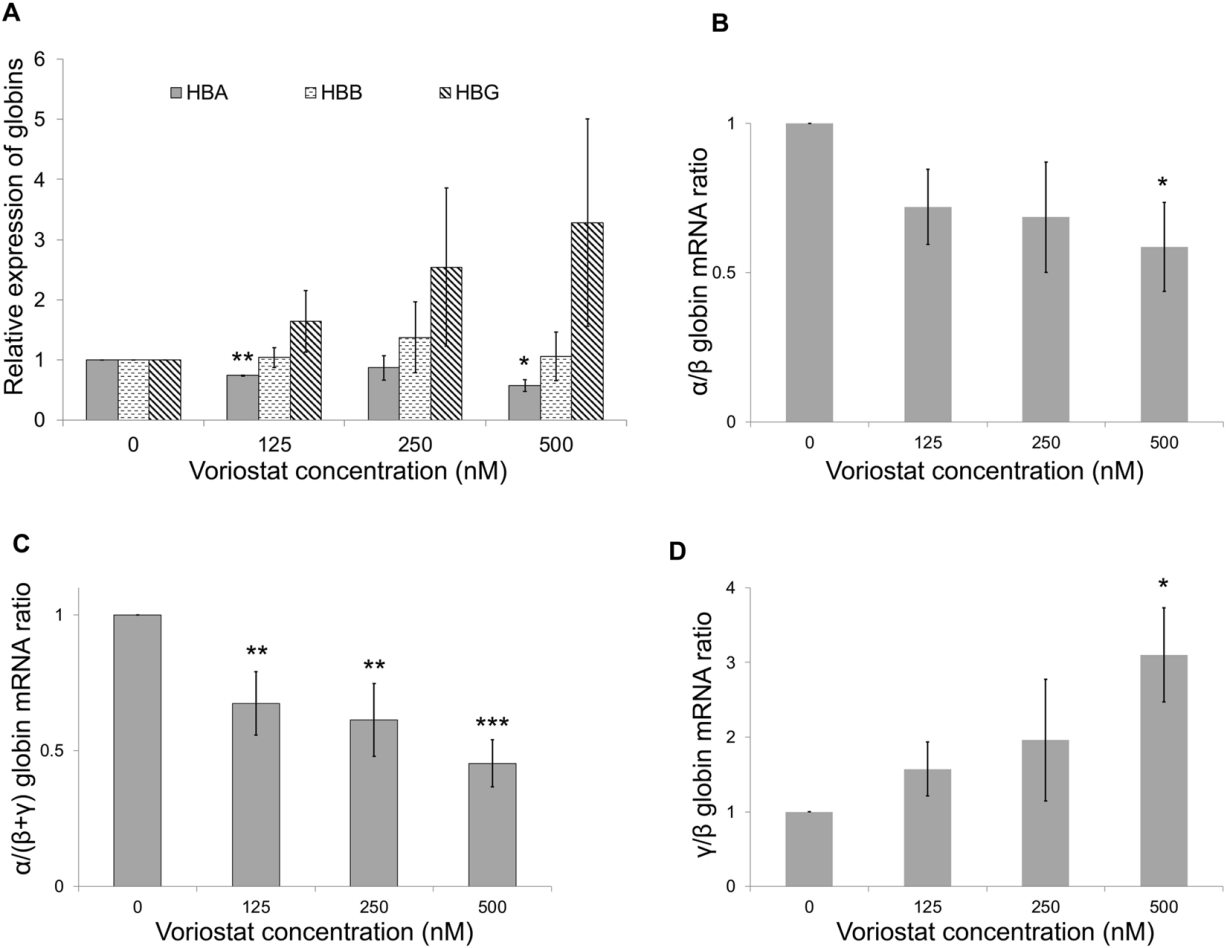
Effects of vorinostat on globin gene expression in human erythroid cells. Erythroid cells differentiated from human adult CD34^+^ cells were incubated with a dose range (0–500 nM) of vorinostat for 72 hours on day 7 of the differentiation and globin gene expression was analysed using qRTPCR. (**A**) α, β and γ-globin expression relative to RPL13A expression. (**B**) α/β-globin mRNA ratio. (**C**) α/(β + γ) globin mRNA ratio. (**D**) γ/β-globin mRNA ratio. Mean expression levels from 3 independent biological repeats are shown; error bars represent SD; *p < 0.05, **p < 0.01 and ***p < 0.001 relative to the 0 nM concentration.

We then evaluated the effect of a concentration gradient of vorinostat in globin gene expression in human erythroid cells using more sensitive qRTPCR platform (Fig. 2A). At nanomolar (125–500 nM) concentrations, vorinostat demonstrated a dose-dependent decrease in α-globin expression in primary human erythroid cells. The expression of β-globin was unaltered, but that of γ-globin was up-regulated in a dose-dependent manner. Furthermore, vorinostat resulted in statistically significant and dose dependent decreases in α/β- and α/(β + γ) globin mRNA ratios (Fig. 2B,C). Also, as expected, vorinostat produced up-regulation of the γ/β globin mRNA ratio (Fig. 2D). Overall, these findings suggest, that in addition to its inducing effects on γ-globin, vorinostat suppresses the expression of α-globin relative to β-like globins, which provides synergistic benefit to patients with β-thalassaemia.

**Figure 3:**
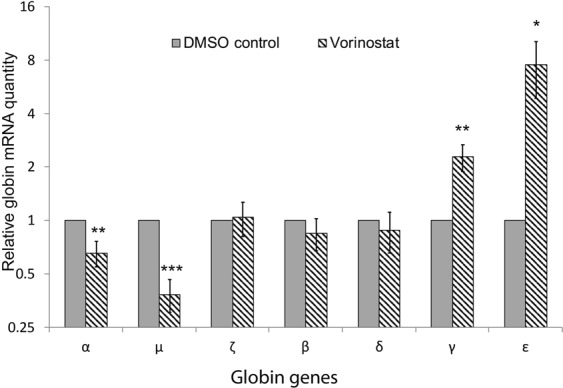
Nanostring quantification of globin gene expression in erythroid cells after treatment with vorinostat. Erythroid cells differentiated from human adult CD34^+^ cells were incubated with vorinostat (500 nM) or DMSO (control) for 72 hours on day 7 of the differentiation and the globin mRNA levels were quantified using Nanostring nCounter digital analyser. Mean Nanostring counts of globin genes normalized to the mean of multiple housekeeping genes (*RPL13A, RPL18, GAPDH, PABPC1, CA2, FTH1, PAIP2 and LAPTM4A*) from 3 independent biological repeats is shown; error bars represent SD; *p < 0.05, **p < 0.01, ***p < 0.001 relative to DMSO control. Scale of the y-axis is logarithmic (base-2).

These observations were verified by the nCounter Digital Analyzer (NanoString Technologies) which provides a measure of absolute number of mRNA molecules without reverse transcription or amplification. Total mRNA quantification using this more sensitive assay confirmed that treatment with vorinostat resulted in a significant reduction in α-globin expression by about 35% in erythroid cells (Fig. 3). γ-globin was up-regulated 2-fold and the expression of β-globin was unaltered. Expression levels of ε-globin demonstrated 7-fold increase in the vorinostat treated cells however, the absolute amounts of mRNA molecules were very low compared to γ- and β-globin (Supplimetal Table 2).

### Effects of vorinostat on erythroid cell proliferation and viability

**Figure 4:**
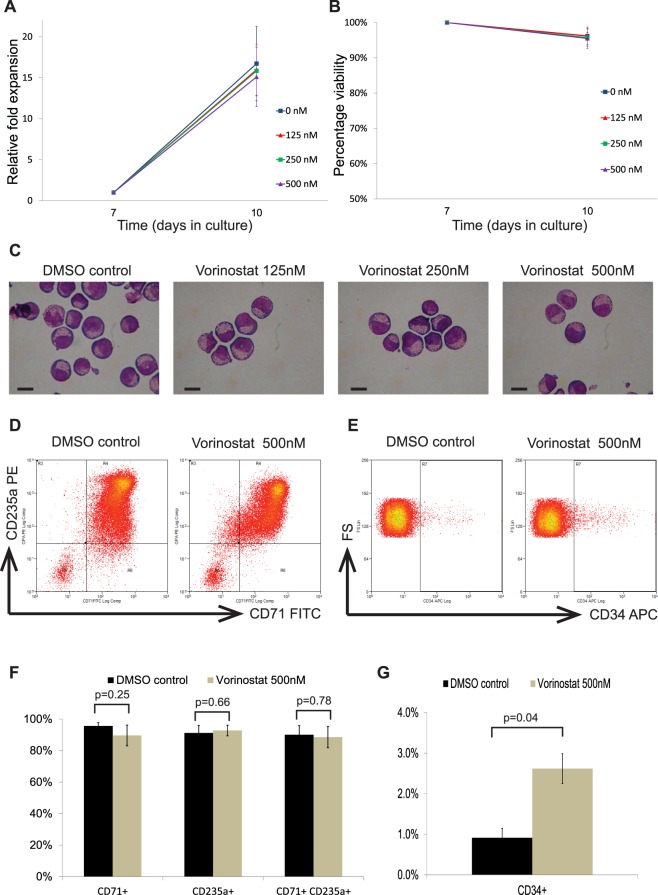
Effects of vorinostat on erythroid cell expansion, viability and differentiation. Erythroid cells differentiated from human umbilical cord blood derived CD34^+^ cells were incubated with a dose range (0–500 nM) of vorinostat for 72 hours on day 7 of the differentiation and analysed on day 10. The 0 nM concentration refers to a DMSO control. (**A**) Cell growth shown as mean fold expansion relative to the number of cells on day 7. (**B**) Percentages of viable cells analysed by trypan blue test. (**C**) Representative cytospins of cells stained by modified Wright stain; scale bar – 10 μm. (**D**) Representative flow cytometry plots of cells stained with FITC-conjugated anti-CD71 and PE-conjugated anti-CD235a antibodies. (**E**) Representative flow cytometry plots of cells stained with APC-conjugated anti-CD34 plotted against forward scatter (FS). (**F**) Percentages of cells expressing CD71 and CD235a in vorinostat treated and control groups. (**G**) Percentage of cells expressing CD34 in vorinostat treated and control groups. In (**A**,**B**) and (**F**,**G**) means of 3 independent biological repeats are shown; error bars represent SD.

Next, we evaluated the effects of vorinostat on erythroid cell proliferation and viability. At the concentrations (125–500 nM) tested, vorinostat did not produce significant effects on cell proliferation and the fold expansions were similar to those of controls (Fig. 4A). Similarly, no significant differences in cellular viability was observed between cells treated with vorinostat and controls (Fig. 4B). Then we assessed the effect of vorinostat on erythroid differentiation *in vitro*. We found that vorinostat did not have significant effects on the stages of erythroid differentiation in the doses tested, and the morphology of cells was similar to control cells at the end of the treatment phase (day 10 of culture) (Fig. 4C). However, immunophenotypical analysis showed that, treatment with vorinostat (500 nM) had minor effects on the expression of some erythroid cell surface markers (Fig. 4D–G). The proportions of cells positive for CD71 and double positive for CD71 and CD235a were not significantly different in the vorinostat treated group compared to the controls (Fig. 4D,F). By contrast, the expression of CD34, an early progenitor cell surface marker, was significantly higher (2.6% vs 0.9%, p < 0.05) in the vorinostat treated group (Fig. 4E,G). Overall, these findings suggest that vorinostat does not have significant effects on erythroid expansion, viability or erythroid differentiation *in vitro*.

### Effects of vorinostat on erythroid transcriptome

**Figure 5:**
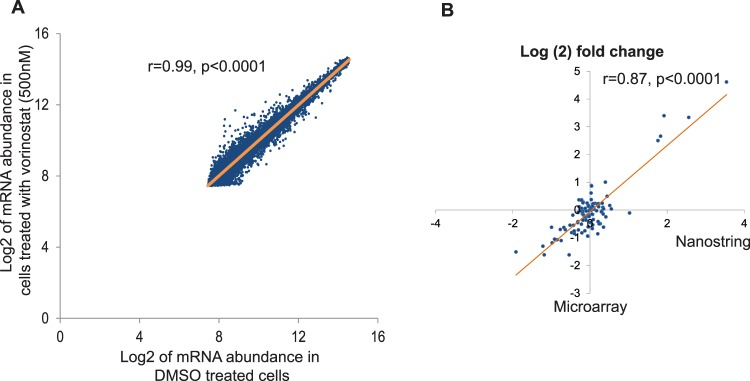
Effect of vorinostat on the erythroid transcriptome. Erythroid cells differentiated from human adult CD34^+^ cells were incubated with vorinostat (500 nM) for 72 hours on day 7 and the microarray analysis was performed on day 10 of erythroid differentiation (n = 3). Low expressing genes were filtered out when comparing the expression levels. (**A**) Scatter plot of Log_2_ mRNA abundance of all the transcripts in vorinostat or DMSO treated cells demonstrating very high (r = 0.99) and statistically significant (p < 0.0001) correlations. (**B**) Validation of microarray data by nanostring; mRNA fold changes of several genes in the same RNA samples used in microarray were independently quantified by nanostring; high correlation (r = 0.87) between data sets validates microarray.

Finally, we tested the effects of vorinostat on global erythroid gene expression using a whole genome gene expression microarray. This microarray, which assayed over 47,000 transcripts revealed that the, mRNA abundance of most of the genes were similar in vorinostat treated cells when compared to controls with a very high correlation coefficient (r = 0.99) (Fig. 5). Two-hundred and four transcripts were found to be differentially regulated between the two groups; 128 (0.3%) transcripts were up-regulated, whereas 76 (0.2%) were down-regulated in vorinostat treated cells (Supplemental Tables 3, 4). Next, we analysed the expression levels of 52 genes which are essential for erythroid physiology (adopted from the publicly available online database, Hembase - http://hembase.niddk.nih.gov/). Expression levels of these genes were not significantly different in vorinostat treated and untreated cells further confirming that vorinostat does not have significant effects on erythroid physiology (Supplemental Table 5).

## Discussion

Histone deacetylation is a recognised epigenetic mechanism that plays a central role in the repression of γ-globin through modifications of associated chromatin and transcription factor activity in the human β-globin locus^20,21^. Inhibition of HDACs, more precisely, HDAC1 and HDAC2 has been shown to induce γ-globin and HbF in a number of *in vitro* studies^22–24^. Therefore, HDAC inhibition has recently been considered as a promising therapeutic option for β-thalassaemia and sickle cell disease (SCD)^13^.

Vorinostat (also known as suberoylanilide hydroxamic acid or SAHA) is a hydroxamic acid-group pan-HDAC inhibitor and is one of the first epigenetic therapies approved for clinical use. It is currently licenced for treatment of advanced cutaneous T-cell lymphoma and is being investigated in clinical trials for number of haematological malignancies including multiple myeloma, acute myeloid leukaemia and myelodysplastic syndrome^25,26^. As for other HDAC inhibitors, vorinostat is a potent inducer of γ-globin and HbF therefore, is also being considered as a potential therapy for SCD and β-thalassaemia^13^. However, its effect on the expression of α-globin has not been identified before. Here, we show that vorinostat also has the ability to down-regulate α-globin expression, making it a potentially ideal candidate to treat β-thalassaemia. Previously, we have shown that the optimal level of reduction of α-globin to produce clinically significant beneficial effects to patients with β-thalassaemia ranges between 25–50%^12^. Importantly, vorinostat decreases the expression of α-globin to these optimal levels (by approximately 35%) increasing its usefulness further. In our assays, vorinostat demonstrated a dose-dependent down-regulation of α-globin expression at low nanomolar concentrations without significant changes in erythroid cell growth or viability and with only mild changes in erythroid differentiation.

Based on its potential to induce HbF, vorinostat was recently tested in a phase 1/2 clinical trial in patients with SCD (www.clinicaltrials.gov #NCT01000155)^27^. This trial which evaluated the efficacy and safety of vorinostat in five patients with SCD concluded that only one out of five patients demonstrated significant induction of HbF at the doses tested however, the drug was safe and tolerable. In this trial the investigators assessed the HbF levels and mRNA levels of β, γ and ε-globin in blood during treatment of vorinostat but did not monitor the expression levels of α-globin. Undoubtedly, in a clinical trial testing vorinostat in the future, it will be extremely useful to include analysis of α-globin mRNA levels in blood as an outcome measure.

Previously, Mai *et al*. have shown that HDAC inhibitors have the potential to down regulate α-globin expression^28^. In this study, the investigators evaluated the effects of two undisclosed HDAC inhibitors on the expression of α-, β- and γ-globin in normal erythroid cells and in cells from two β-thalassaemia patients. Following treatment with one of the HDAC inhibitor, cells derived from one patient showed a dose dependent decrease in α-globin expression whereas the cells from the other patient did not. This observation suggest that there may be still unknown genetic and biological factors that alter the response to treatment with HDAC inhibitors which may explain the variability of the clinical response to vorinostat in the above clinical trial.

Vorinostat has several advantages as a potential therapy for β-thalassaemia. It is already a licenced medication for other indications with an available oral dosage form, of which, the safety, tolerability and clinical pharmacology profile have been characterised^29,30^. Therefore, it could be used in human subjects and patients without the necessity for additional pre-clinical or animal studies. In addition, vorinostat would benefit patients with β-thalassaemia through two independent pathways – direct silencing of α-globin and induction of γ-globin – which act synergistically to reduce the excess free α-globin chains in RBCs; patients with haemoglobin E β-thalassaemia seem to be particularly sensitive to a reduction in α-globin expression^12^.

The mechanism by which vorinostat exerts these beneficial effects on globin gene expression in human erythroid cells is not well understood yet. Although, γ-globin induction is most likely to be due to inhibition of HDAC, silencing of α-globin may be through a different or an indirect pathway. Interestingly, there is evidence to suggest that vorinostat has inhibitory activity against histone demethylases (KDMs) in addition to HDACs^31,32^. As histone methylation is a recognised epigenetic mechanism regulating α-globin^12^ and the broad-range KDM inhibitor, IOX1, down-regulates α-globin expression^17^, it is possible that the α-globin down-regulation by vorinostat is mediated through inhibition of histone demethylases.

Although vorinostat does not have significant effects on the erythroid differentiation in general, the expression levels of CD34, was significantly higher in the vorinostat treated group. This finding is in consistent with previous observations which demonstrated higher expansions of CD34^+^ cells in cord blood haematopoietic stem cells treated with HDAC inhibitors^33^.

One limitation of our study is that due to restrictions in available assays we did not evaluate the effect of vorinostat at globin protein level. However, the mechanism of action of vorinostat is already known to be through inhibition of HDAC and alteration of epigenetic environment which exerts its effects at the level of gene expression. Therefore, it is reasonable to postulate that the globin mRNA changes that we observed will be translated into protein level as well. Additionally, as vorinostat is already an FDA approved drug, differential globin protein levels can be quantified in a future clinical trial on patients with β-thalassaemia.

In conclusion, we have demonstrated that the US FDA approved HDAC inhibitor vorinostat down-regulates α-globin expression whilst inducing γ-globin expression in human erythroid cells and is a potential therapy for β-thalassaemia. This new finding would strengthen the knowledge base of this epigenetic drug, which has already made significant progress into the clinical trials towards treatment of β-haemoglobinopathies.

## Methods

### Human CD34+ erythroid differentiation culture

Human umbilical cord blood units and peripheral blood leucocyte cones were purchased from the National Health Service Blood and Transplant (NHSBT), UK. Ethical approval for the study was granted by the North West Research Ethics Committee of NHS National Research Ethics Services, UK (reference No. 03/08/097). Human CD34+ hematopoietic stem and progenitor cells (HSPC) were purified using CD34 MicroBead Kit (Miltenyl Biotech) and were cultured in a previously described two-phase liquid culture system^34^. In brief, CD34+ cells were cultured in StemSpan serum free expansion medium supplemented with 100 ng/ml stem cell factor, 10 ng/ml interleukin-3, 10ug/ml cholesterol rich lipids and 0.5IU/ml erythropoietin in the phase 1. After 7 days, cells were transferred into phase 2 differentiation medium, which is similar to phase 1 medium except for the addition of 0.5 mg/ml iron saturated holotransferrin and higher concentration of erythropoietin (3U/ml). Throughout the culture, cells were maintained in a 5% CO_2_ atmosphere at 37 °C and the cell concentration was kept below 2 million/ml by adding fresh medium every 2–3 days.

### Treatment with vorinostat

Cells were incubated for 72 hours from day 7 to 10 of the erythroid culture with different concentrations of vorinostat (purchased from Sigma; Cat No: SML0061).

### Cellular morphology and flow cytometry

Cell viability was determined using trypan blue test whilst morphology was assessed using cytospins stained with modified Wright stain. For flow cytometry, washed cells were labelled for 20 minutes on ice, in 2% bovine serum albumin, with the following monoclonal anti-human antibodies; allophycocyanin (APC) conjugated anti-CD34 (Miltenyl Biotech), fluorescein isothiocyanate (FITC) conjugated anti-CD71 (BD Pharmingen) and phycoerythrin (PE) conjugated anti-glycophorin A (BD Pharmingen). Analysis was performed on a CyanTM ADP analyzer using Summit v4.3 software after gating on viable cells identified with a Hoechst 33258 pentahydrate (Invitrogen) nucleic acid stain.

### RNA extraction, reverse transcription and gene expression analysis

Total RNA was purified using the RNeasy mini kit (Qiagen) and complementary DNA (cDNA) was prepared using the high capacity RNA to cDNA kit (Applied Biosystems). Quantitative polymerase chain reaction (qPCR) was performed in a 7500 fast real time PCR system (Applied Biosystems) according to the manufacturer’s protocol using validated, inventoried and exon-spanning TaqMan assays (Applied Biosystems) for human α-globin, β-globin, γ-globin and ribosomal protein L13A (*RPL13A*). Data were analyzed by 7500 software v2.0.6 using the delta delta CT method. *RPL13A* was used as the house keeping gene for normalizations.

For Nanostring experiments, we purchased a custom-made capture probe set panel and consumables from Nanostring technologies. Hybridized samples were processed using the nCounter prep station and nCounter digital analyzer (Nanostring Technologies) according to the manufacturer’s instructions. Raw data were normalized to an internal positive spike-in control to normalize to all of the platform’s associated sources of variation and then to the geometric mean of eight housekeeping genes (*RPL13A, RPL18, GAPDH, PABPC1, CA2, FTH1, PAIP2* and *LAPTM4A*).

### Microarray

Microarray whole genome gene expression analysis was performed using Illumina’s Human HT12v4.0 Expression BeadChip and Illumina iScan Scanner. The experiments were performed in biological triplicate and the data were normalized in R using the lumi package, analysed using Linear Models for Microarray (limma) and filtered using an adjusted P value of < 0.05 to identify differentially expressed genes. Low expressing genes were filtered out when comparing the expression levels of genes in vorinostat treated and untreated cells.

### Statistical analysis

A two-tailed unpaired Student’s t-test was used in statistical analysis between groups for normally distributed data. Differences corresponding to p < 0.05 were considered statistically significant.

### Ethics statement

All experiments were carried out in accordance with Declaration of Helsinki and were approved by the University of Oxford and University of Kelaniya. Ethical approval for the study was granted by the North West Research Ethics Committee of NHS National Research Ethics Services, UK (reference No. 03/08/097) and the Ethics Review Committee of the University of Kelaniya (reference No. P/71/02/2018). Informed consent to use human samples for research were obtained from the participants by the providers of cells (NHSBT, UK).

## Supplementary information

Supplementary Data File
