# Supplementary material for: Synergistic silencing of α-globin and induction of γ-globin by histone deacetylase inhibitor, vorinostat as a potential therapy for β-thalassaemia
Source: Sci Rep. 2019 Aug 12;9:11649. doi: 10.1038/s41598-019-48204-2 (PMC6690964; doi:10.1038/s41598-019-48204-2)
Supplement: Supplementary file 1 — Supplementary Data File [file 41598_2019_48204_MOESM1_ESM.docx]

**Supplementary Data File**

# Article title: Synergistic silencing of α-globin and induction of γ-globin by histone deacetylase inhibitor, vorinostat as a potential therapy for β-thalassaemia

# Author List: Sachith Mettananda, Nirmani Yasara, Christopher A. Fisher, Stephen Taylor, Richard Gibbons, Doug Higgs

**Supplemental table 1 – List of HDAC drugs/compounds in the epigenetic inhibitor small molecule library**

| Small molecule inhibitor | Inhibitor target/class | Working concentration (µM) |
| --- | --- | --- |
| CXD101 | HDAC | 1 |
| Entinostat (MS-275) | HDAC -ortho-amino anilides | 0.5 |
| Trichostatin A | HDAC -hydroxamic acid Class I & 2 | 0.5 |
| Vorinostat | HDAC -hydroxamic acid | 2.5 |
| Valproic acid | HDAC -aliphatic acid compounds | 1000 |

**Supplemental table 2 – Normalised Nanostring counts of globin genes in vorinostat treated and control cells^1^**

| ***Globin gene*** | ***Mean (±SD) Nanostring counts*** | | ***Fold change*** |
| --- | --- | --- | --- |
|  | ***DMSO Control*** | ***Vorinostat 500nM*** |  |
| α-globin | 5490 (±1118) | 3517 (±240) | 0.64 |
| μ-globin | 598 (±222) | 218 (±48) | 0.36 |
| ζ−globin | 23 (±4.6) | 23 (±1.1) | 1.01 |
| β-globin | 37768 (±7451) | 31405 (±3798) | 0.83 |
| δ-globin | 11623 (±3203) | 9802 (±931) | 0.84 |
| γ-globin | 25351 (±5411) | 56609 (±9000) | 2.23 |
| ε-globin | 24 (±24) | 160 (±125) | 6.67 |

**^1^**Erythroid cells differentiated from human adult CD34^+^ cells were incubated with vorinostat (500nM) or DMSO (control) for 72 hours on day 7 of the differentiation and the globin mRNA levels were quantified using Nanostring nCounter digital analyser. Mean (±standard deviation) Nanostring counts of globin genes normalized to the mean of multiple housekeeping genes (*RPL13A, RPL18, GAPDH, PABPC1, CA2, FTH1, PAIP2 and LAPTM4A*) from 3 independent biological repeats are shown.

Supplemental table 3 – List of genes significantly up-regulated in vorinostat treated cells (in alphabetical order)

| Gene Symbol | Gene Name | Fold change |
| --- | --- | --- |
| ABCB1 | ATP-binding cassette, sub-family B | 1.39 |
| ABHD6 | Abhydrolase domain containing 6 | 2.07 |
| ALOX5AP | Arachidonate 5-lipoxygenase-activating protein | 2.07 |
| ANXA1 | Annexin A1 | 3.31 |
| AP1S2 | Adaptor-related protein complex 1, sigma 2 subunit | 2.00 |
| APOE | Apolipoprotein E | 2.09 |
| ARRB1 | Beta 1 | 1.68 |
| ATP2A3 | ATPase, Ca++ transporting, ubiquitous | 1.89 |
| BEND4 | BEN domain containing 4 | 1.29 |
| BEX1 | Brain expressed, X-linked 1 | 2.85 |
| C11ORF60 | Chromosome 11 open reading frame 60 | 1.36 |
| C12ORF45 | Chromosome 12 open reading frame 45 | 1.50 |
| C14ORF112 | Chromosome 14 open reading frame 112 | 1.40 |
| C15ORF39 | Chromosome 15 open reading frame 39 | 1.99 |
| C16ORF45 | Chromosome 16 open reading frame 45 | 1.31 |
| C1ORF183 | Chromosome 1 open reading frame 183 | 1.25 |
| C20ORF3 | Chromosome 20 open reading frame 3 | 1.56 |
| C21ORF63 | Chromosome 21 open reading frame 63 | 1.49 |
| C2ORF18 | Chromosome 2 open reading frame 18 | 1.53 |
| CBX6 | Chromobox homolog 6 | 1.87 |
| CCND2 | Cyclin D2 | 2.15 |
| CD52 | CD52 molecule | 4.17 |
| CD53 | CD53 molecule | 1.67 |
| CD74 | CD74 molecule | 7.26 |
| CENPBD1 | CENPB DNA-binding domains containing 1 | 1.32 |
| CHD7 | Chromodomain helicase DNA binding protein 7 | 1.68 |
| CHST7 | Carbohydrate (N-acetylglucosamine 6-O) sulfotransferase 7 | 1.41 |
| CLDN10 | Claudin 10 | 1.94 |
| CLDN11 | Claudin 11 | 3.09 |
| CORO1A | Coronin, actin binding protein, 1A | 1.70 |
| CSF2RB | Colony stimulating factor 2 receptor, beta | 1.45 |
| CUGBP2 | CUG triplet repeat, RNA binding protein 2 | 1.99 |
| CYFIP2 | Cytoplasmic FMR1 interacting protein 2 | 1.70 |
| DBI | Diazepam binding inhibitor | 1.54 |
| DEPDC7 | DEP domain containing 7 | 1.25 |
| EIF3E | Eukaryotic translation initiation factor 3, subunit E | 1.21 |
| EPB41L2 | Erythrocyte membrane protein band 4.1-like 2 | 1.69 |
| EXTL2 | Exostoses (multiple)-like 2 | 1.53 |
| FADS1 | Fatty acid desaturase 1 | 1.42 |
| FAM190B | Family with sequence similarity 190, member B | 1.23 |
| FAM27A | Family with sequence similarity 27, member A | 1.40 |
| FCGR2A | Fc fragment of IgG, low affinity IIa, receptor | 1.90 |
| FKSG30 | Actin-like protein | 1.83 |
| GALM | Galactose mutarotase (aldose 1-epimerase) | 1.84 |
| HLA-A | Major histocompatibility complex, class I, A | 2.31 |
| HLA-B | Major histocompatibility complex, class I, B | 2.64 |
| HLA-DMA | Major histocompatibility complex, class II, DM alpha | 3.27 |
| HLA-DPA1 | Major histocompatibility complex, class II, DP alpha 1 | 4.54 |
| HLA-DRA | Major histocompatibility complex, class II, DR alpha | 7.40 |
| HLA-DRB1 | Major histocompatibility complex, class II, DR beta 1 | 2.21 |
| HLA-DRB6 | Major histocompatibility complex, class II, DR beta 6 (pseudogene) | 1.98 |
| HS.133181 | Soares_parathyroid_tumor_NbHPA | 1.62 |
| HS.193406 | cDNA FLJ34755 fis | 1.66 |
| ID2 | Inhibitor of DNA binding 2 | 2.10 |
| IGSF3 | Immunoglobulin superfamily, member 3 | 1.72 |
| IL1B | Interleukin 1, beta | 2.30 |
| ITM2C | Integral membrane protein 2C | 1.81 |
| KIAA1522 | KIAA1522 | 1.56 |
| KLHL5 | Kelch-like 5 (Drosophila) | 1.90 |
| LCP1 | Lymphocyte cytosolic protein 1 (L-plastin) | 2.17 |
| LEF1 | Lymphoid enhancer-binding factor 1 | 1.83 |
| LIMA1 | LIM domain and actin binding 1 | 1.33 |
| LITAF | Lipopolysaccharide-induced TNF factor | 3.42 |
| LOC100132948 | Similar to Protein FAM27A/B/C, transcript variant 1 | 1.54 |
| LOC401076 | Misc_RNA | 1.31 |
| LOC606724 | Actin binding protein, 1A pseudogene | 1.70 |
| LOC643319 | Similar to Transgelin-2 | 1.51 |
| LOC644760 | Hypothetical protein LOC644760 | 2.38 |
| LOC645553 | Hypothetical LOC645553 | 2.56 |
| LOC730415 | Hypothetical LOC730415, transcript variant 2 | 3.94 |
| LRRC20 | Leucine rich repeat containing 20 | 1.56 |
| LY6E | Lymphocyte antigen 6 complex, locus E | 2.41 |
| LYN | Yamaguchi sarcoma viral related oncogene homolog | 1.70 |
| MAP3K7 | Mitogen-activated protein kinase kinase kinase 7 | 1.33 |
| MEF2C | Myocyte enhancer factor 2C | 1.50 |
| MFSD1 | Major facilitator superfamily domain containing 1 | 1.45 |
| MGC71993 | Similar to DNA segment, Chr 11, | 1.54 |
| MLLT11 | Myeloid/lymphoid or mixed-lineage leukemia | 2.37 |
| MSN | Moesin | 2.31 |
| MTM1 | Myotubularin 1 | 1.28 |
| MYC | Myelocytomatosis viral oncogene homolog | 1.62 |
| NME7 | Non-metastatic cells 7, protein | 1.47 |
| NUDT11 | Nudix (nucleoside diphosphate linked moiety X)-type motif 11 | 1.67 |
| P76 | Mannose-6-phosphate protein p76 | 1.35 |
| PA2G4 | Proliferation-associated 2G4 | 1.49 |
| PAQR8 | Progestin and adipoQ receptor family member VIII | 1.40 |
| PELI2 | Pellino homolog 2 (Drosophila) | 1.82 |
| PHC1 | Polyhomeotic homolog 1 (Drosophila) | 1.31 |
| PNKD | Paroxysmal nonkinesiogenic dyskinesia | 1.73 |
| POU4F1 | POU class 4 homeobox 1 | 1.69 |
| PRKCB | Protein kinase C, beta | 3.02 |
| PRKCB1 | Protein kinase C, beta 1 | 3.50 |
| PRNP | Prion protein | 1.45 |
| PSMB9 | Proteasome (prosome, macropain) subunit, beta type, 9 | 1.51 |
| PTDSS1 | Phosphatidylserine synthase 1 | 1.36 |
| RAB11FIP5 | RAB11 family interacting protein 5 (class I) | 1.53 |
| RAB31 | RAB31, member RAS oncogene family | 2.66 |
| RAB37 | RAB37, member RAS oncogene family | 1.72 |
| RAB38 | RAB38, member RAS oncogene family | 2.44 |
| RNASEK | Ribonuclease, RNase K | 1.82 |
| RRAGD | Ras-related GTP binding D | 1.46 |
| SAMSN1 | SAM domain, SH3 domain and nuclear localization signals 1 | 1.98 |
| SDPR | Serum deprivation response (phosphatidylserine binding protein) | 2.32 |
| SEPT6 | Septin 6 | 1.46 |
| SKAP1 | Src kinase associated phosphoprotein 1 | 2.18 |
| SLC16A10 | Solute carrier family 16, member 10 | 1.61 |
| SLC27A5 | Solute carrier family 27 | 1.64 |
| SLC44A1 | Solute carrier family 44, member 1 | 1.72 |
| SLC8A3 | Solute carrier family 8 , member 3 | 1.72 |
| SNPH | Syntaphilin | 1.34 |
| SPI1 | Spleen focus forming virus proviral integration oncogene spi1 | 1.64 |
| STAT3 | Signal transducer and activator of transcription 3 | 1.70 |
| STXBP5 | Syntaxin binding protein 5 | 2.09 |
| SWAP70 | SWAP switching B-cell complex 70kDa subunit | 1.65 |
| TAC3 | Tachykinin 3 | 2.16 |
| TCEAL8 | Transcription elongation factor A (SII)-like 8 | 1.59 |
| TCN1 | Transcobalamin I | 2.25 |
| TMEM206 | Transmembrane protein 206 | 1.48 |
| TMSL3 | Thymosin-like 3 | 1.70 |
| TRDMT1 | tRNA aspartic acid methyltransferase 1 | 1.26 |
| TSC22D1 | TSC22 domain family, member 1 | 1.55 |
| TSPAN33 | Tetraspanin 33 | 2.06 |
| TUBA1A | Tubulin, alpha 1a | 1.99 |
| TUBB4 | Tubulin, beta 4 | 1.35 |
| TUFM | Tu translation elongation factor, mitochondrial | 1.44 |
| VAT1 | Vesicle amine transport protein 1 homolog | 2.02 |
| WBP5 | WW domain binding protein 5 | 2.26 |
| ZFP36L2 | Zinc finger protein 36, C3H type-like 2 | 1.89 |

Supplemental table 4 – List of genes significantly down-regulated in vorinostat treated cells (in alphabetical order)

| Gene Symbol | Gene Name | Fold change |
| --- | --- | --- |
| ADD2 | Adducin 2 (beta) | 0.61 |
| AFG3L2 | AFG3 ATPase family gene 3-like 2 (yeast) | 0.70 |
| ANKRD9 | Ankyrin repeat domain 9 | 0.43 |
| ASNS | Asparagine synthetase | 0.49 |
| ASPSCR1 | Alveolar soft part sarcoma chromosome region | 0.61 |
| ATF4 | Activating transcription factor 4 | 0.72 |
| ATF5 | Activating transcription factor 5 | 0.30 |
| B9D2 | B9 protein domain 2 | 0.72 |
| BTBD6 | BTB (POZ) domain containing 6 | 0.49 |
| C12ORF24 | Chromosome 12 open reading frame 24 | 0.72 |
| CCDC121 | Coiled-coil domain containing 121 | 0.77 |
| CENPM | Centromere protein M | 0.75 |
| CENPV | Centromere protein V | 0.66 |
| CLCN3 | Chloride channel 3 | 0.72 |
| CREBBP | CREB binding protein | 0.74 |
| DDX59 | DEAD (Asp-Glu-Ala-Asp) box polypeptide 59 | 0.55 |
| DNAJB2 | DnaJ (Hsp40) homolog, subfamily B, member 2 | 0.57 |
| DOCK7 | Dedicator of cytokinesis 7 | 0.67 |
| EIF4EBP3 | Eukaryotic translation initiation factor 4E binding protein 3 | 0.66 |
| EPCAM | Epithelial cell adhesion molecule | 0.45 |
| EPRS | Glutamyl-prolyl-tRNA synthetase | 0.68 |
| FBXL20 | F-box and leucine-rich repeat protein 20 | 0.75 |
| FGFR3 | Fibroblast growth factor receptor 3 | 0.56 |
| FOXRED2 | FAD-dependent oxidoreductase domain containing 2 | 0.74 |
| FREQ | Frequenin homolog (Drosophila) | 0.42 |
| GOLSYN | Golgi-localized protein | 0.69 |
| GPR137 | G protein-coupled receptor 137 | 0.64 |
| GTF2IRD2B | GTF2I repeat domain containing 2B | 0.59 |
| HS.352549 | cDNA clone IMAGE:30406177 5, mRNA sequence | 0.69 |
| IL18BP | Interleukin 18 binding protein | 0.61 |
| INO80D | INO80 complex subunit D | 0.71 |
| KAT2B | K(lysine) acetyltransferase 2B | 0.53 |
| KBTBD2 | Kelch repeat and BTB (POZ) domain containing 2 | 0.72 |
| KLHL22 | Kelch-like 22 (Drosophila) | 0.69 |
| LIME1 | Lck interacting transmembrane adaptor 1 | 0.61 |
| LOC440498 | Hypothetical gene supported by AK001829 | 0.67 |
| LOC643008 | PP12104, transcript variant 1 | 0.58 |
| LOC729446 | Smilar to AT rich interactive domain 1B (SWI1-like) isoform 1 | 0.73 |
| LOC729920 | Notch1-induced protein | 0.80 |
| MESP1 | Mesoderm posterior 1 homolog (mouse) | 0.66 |
| MKLN1 | Muskelin 1, intracellular mediator containing kelch motifs | 0.68 |
| MKNK2 | MAP kinase interacting serine/threonine kinase 2 | 0.52 |
| MST1 | Macrophage stimulating 1 | 0.43 |
| MTX1 | Metaxin 1 | 0.67 |
| MUC1 | Mucin 1 | 0.36 |
| NT5DC1 | 5'-nucleotidase domain containing 1 | 0.75 |
| OPLAH | 5-oxoprolinase | 0.54 |
| PARP3 | Poly (ADP-ribose) polymerase family, member 3 | 0.64 |
| PCK2 | Phosphoenolpyruvate carboxykinase 2 | 0.57 |
| PEX19 | Peroxisomal biogenesis factor 19 | 0.73 |
| PHF2 | PHD finger protein 2 | 0.73 |
| PHPT1 | Phosphohistidine phosphatase 1 | 0.69 |
| PLEKHH3 | Pleckstrin homology domain containing, family H member 3 | 0.54 |
| PMM1 | Phosphomannomutase 1 | 0.55 |
| POLE4 | Polymerase (DNA-directed), epsilon 4 (p12 subunit) | 0.57 |
| PQLC1 | PQ loop repeat containing 1 | 0.52 |
| PSAT1 | Phosphoserine aminotransferase 1 | 0.34 |
| PSMB10 | Proteasome (prosome, macropain) subunit, beta type, 10 | 0.69 |
| RCN1 | Reticulocalbin 1, EF-hand calcium binding domain | 0.50 |
| SFMBT1 | Scm-like with four mbt domains 1 | 0.78 |
| SIX5 | SIX homeobox 5 | 0.74 |
| SLC11A2 | Solute carrier family 11 , member 2 | 0.65 |
| SLC2A4RG | SLC2A4 regulator | 0.61 |
| SLC3A2 | Solute carrier family , member 2 | 0.55 |
| STAU1 | RNA binding protein, homolog 1 (Drosophila) | 0.76 |
| TACSTD1 | Tumor-associated calcium signal transducer 1 | 0.39 |
| TMEM18 | Transmembrane protein 18 | 0.70 |
| TMEM87A | Transmembrane protein 87A | 0.71 |
| TMPRSS9 | Transmembrane protease, serine 9 | 0.83 |
| TPM1 | Tropomyosin 1 (alpha) | 0.66 |
| TRMT5 | TRM5 tRNA methyltransferase 5 homolog (S. cerevisiae) | 0.72 |
| TXNRD2 | Thioredoxin reductase 2 | 0.50 |
| UFC1 | Ubiquitin-fold modifier conjugating enzyme 1 | 0.61 |
| URG4 | Up-regulated gene 4 | 0.72 |
| VPS37C | Vacuolar protein sorting 37 homolog C (S. cerevisiae) | 0.70 |
| ZNF428 | Zinc finger protein 428 | 0.65 |

Supplemental table 5 – Expression levels of genes essential for erythrocyte physiology in vorinostat treated cells compared to DMSO control cells (n=3)^[[1]](#footnote-1)^

| ***Genes***  ***symbol*** | ***Gene name*** | ***Log_2_ of mRNA abundance*** | | ***Fold change*** |
| --- | --- | --- | --- | --- |
|  |  | ***DMSO*** | ***Vorinostat 500nM*** |  |
| PPOX | Protoporphyrinogen oxidase | 11.47 | 10.54 | 0.53 |
| GSTT1 | Glutathione S-transferase theta 1 | 9.94 | 9.16 | 0.58 |
| TMOD1 | Tropomodulin 1 | 10.29 | 9.56 | 0.60 |
| SLC4A1 | Solute carrier family 4 | 11.49 | 10.82 | 0.63 |
| GPI | Glucose phosphate isomerase | 11.96 | 11.37 | 0.66 |
| GYPB | Glycophorin B | 11.97 | 11.43 | 0.69 |
| PFKM | Phosphofructokinase, muscle | 9.76 | 9.24 | 0.70 |
| UROS | Uroporphyrinogen III synthase | 12.68 | 12.20 | 0.72 |
| TPM1 | Tropomyosin 1 | 11.64 | 11.19 | 0.73 |
| EPB42 | Erythrocyte membrane protein band 4.2 | 12.05 | 11.60 | 0.73 |
| GYPA | Glycophorin A | 10.71 | 10.27 | 0.74 |
| SPTA1 | Spectrin, alpha | 11.63 | 11.24 | 0.77 |
| GYPE | Glycophorin E | 10.30 | 10.01 | 0.82 |
| HMBS | Hydroxymethylbilane synthase | 12.33 | 12.06 | 0.83 |
| TPI1 | Triosephosphate isomerase 1 | 13.56 | 13.30 | 0.83 |
| EPB49 | Erythrocyte membrane protein band 4.9 | 9.95 | 9.75 | 0.87 |
| NT5C3 | 5'-nucleotidase, cytosolic III | 11.42 | 11.23 | 0.88 |
| UROD | Uroporphyrinogen decarboxylase | 13.44 | 13.27 | 0.89 |
| RHD | Rhesus blood group, D antigen | 9.06 | 8.91 | 0.90 |
| PGD | Phosphogluconate dehydrogenase | 11.93 | 11.80 | 0.91 |
| AQP1 | Aquaporin 1 | 7.75 | 7.62 | 0.92 |
| PKLR | Pyruvate kinase, liver and RBC | 11.43 | 11.31 | 0.92 |
| PRDX2 | Peroxiredoxin 2 | 8.00 | 7.90 | 0.94 |
| GSR | Glutathione reductase | 7.72 | 7.65 | 0.95 |
| CPOX | Coproporphyrinogen oxidase | 9.98 | 9.91 | 0.95 |
| ALAD | Aminolevulinate, delta-, dehydratase | 8.09 | 8.03 | 0.96 |
| FECH | Ferrochelatase | 7.83 | 7.76 | 0.96 |
| RHAG | Rhesus blood group-associated glycoprotein | 12.69 | 12.63 | 0.96 |
| G6PD | Glucose-6-phosphate dehydrogenase | 7.69 | 7.65 | 0.97 |
| GYPC | Glycophorin C | 7.62 | 7.61 | 1.00 |
| HK1 | Hexokinase 1 | 10.62 | 10.62 | 1.00 |
| GSS | Glutathione synthetase | 9.97 | 9.98 | 1.01 |
| ALDOA | Aldolase A, fructose-bisphosphate | 7.54 | 7.55 | 1.01 |
| AQP3 | Aquaporin 3 | 8.15 | 8.18 | 1.02 |
| ADD3 | Adducin 3 | 7.52 | 7.55 | 1.02 |
| GCLC | Glutamate-cysteine ligase | 7.55 | 7.59 | 1.02 |
| PGK1 | Phosphoglycerate kinase 1 | 10.34 | 10.39 | 1.04 |
| RHCE | Rhesus blood group, CcEe antigens | 10.86 | 10.93 | 1.05 |
| EPB41 | Erythrocyte membrane protein band 4.1 | 8.65 | 8.74 | 1.06 |
| BPGM | 2,3-bisphosphoglycerate mutase | 8.93 | 9.03 | 1.08 |
| ADD1 | Adducin 1 | 8.88 | 9.01 | 1.09 |
| SPTB | Spectrin, beta | 7.57 | 7.71 | 1.10 |
| ADA | Adenosine deaminase | 9.33 | 9.54 | 1.16 |
| ACTB | Actin, beta | 14.19 | 14.42 | 1.17 |
| ANK1 | Ankyrin 1 | 7.76 | 8.00 | 1.18 |
| GPX1 | Glutathione peroxidase 1 | 11.96 | 12.21 | 1.19 |
| ENO1 | Enolase 1 | 13.83 | 14.08 | 1.19 |
| ALAS2 | Aminolevulinate, delta-, synthase 2 | 8.84 | 9.09 | 1.19 |
| CD47 | CD47 antigen | 10.00 | 10.26 | 1.20 |
| HMOX1 | Heme oxygenase (decycling) 1 | 7.79 | 8.05 | 1.20 |
| LDHB | Lactate dehydrogenase B | 11.74 | 12.04 | 1.24 |
| STOM | Stomatin | 11.89 | 12.20 | 1.24 |

1. The mRNA abundance determined by microarray in cells treated with vorinostat (500nM) and DMSO (control) for 72 hours on day 7 of erythroid differentiation is shown here. The list of genes is adopted from Hembase (http://hembase.niddk.nih.gov/), a database of genes with specific and essential roles in erythrocyte physiology. Fold change represents the fold difference of mRNA abundance between vorinostat and DMSO treated cells. None of the 52 genes was differentially expressed in vorinostat treated cells compared to control. [↑](#footnote-ref-1)
